# Management of Superficial Esophageal Squamous Cell Carcinoma and Early Gastric Cancer following Non-Curative Endoscopic Resection

**DOI:** 10.3390/cancers14153757

**Published:** 2022-08-02

**Authors:** Waku Hatta, Tomoyuki Koike, Kaname Uno, Naoki Asano, Atsushi Masamune

**Affiliations:** Division of Gastroenterology, Tohoku University Graduate School of Medicine, Sendai 980-8574, Japan; waku-style@festa.ocn.ne.jp (W.H.); tkoike@rd5.so-net.ne.jp (T.K.); kaname@wa2.so-net.ne.jp (K.U.); asanon@med.tohoku.ac.jp (N.A.)

**Keywords:** superficial esophageal squamous cell carcinoma, early gastric cancer, non-curative endoscopic resection

## Abstract

**Simple Summary:**

Guidelines recommend additional treatment following non-curative endoscopic resection in cases of superficial esophageal squamous cell carcinoma and early gastric cancer because of the potential risk of lymph node metastasis. This review discusses recent advances in this field, including the development of pathological risk stratification for metastatic recurrence and identification of different recurrence patterns after non-curative endoscopic resection for superficial esophageal squamous cell carcinoma or early gastric cancer, and the establishment of a novel treatment strategy for clinical T1b-SM esophageal squamous cell carcinoma. For optimal therapeutic decision-making in such patients, it is also important to predict prognoses other than superficial esophageal squamous cell carcinoma or early gastric cancer and impaired quality of life. Thus, a novel algorithm that considers these factors and metastatic recurrence is required.

**Abstract:**

According to the European and Japanese guidelines, additional treatment is recommended for cases of superficial esophageal squamous cell carcinoma (ESCC) and early gastric cancer (EGC) that do not meet the curability criteria for endoscopic resection (ER), i.e., non-curative ER, owing to the risk of lymph node metastasis (LNM). However, the rates of LNM in such cases were relatively low (e.g., 8% for EGC). Several recent advances have been made in this field. First, pathological risk stratification for metastatic recurrence following non-curative ER without additional treatment was developed for both superficial ESCC and EGC. Second, the pattern of metastatic recurrence and prognosis after recurrence following non-curative ER without additional treatment was found to be considerably different between superficial ESCC and EGC. Third, a combination of ER and selective chemoradiotherapy was developed as a minimally invasive treatment method for clinical T1b-SM ESCC. These findings may help clinicians decide the treatment strategy for patients following non-curative ER; however, for optimal therapeutic decision-making in such patients, it is also important to predict the prognosis other than SESCC or EGC and impaired quality of life. Thus, a novel algorithm that considers these factors, as well as metastatic recurrence, should be developed.

## 1. Introduction

With the advances in endoscopic technologies, esophageal squamous cell carcinoma (ESCC) and gastric cancer can be detected at an early stage [1,2,3,4]. Endoscopic resection (ER) is now widely performed for superficial ESCC (SESCC) and early gastric cancer (EGC) that are preoperatively diagnosed as having a negligible risk of lymph node metastasis (LNM) [5,6,7,8]. In addition, the introduction of the endoscopic submucosal dissection (ESD) technique has enabled en bloc resection of larger lesions and expanded the indications of ER for SESCC and EGC [9,10]. The use of ESD is prevalent in Eastern Asian countries [11,12,13,14,15], and this technique is now widely performed in Western countries [16,17]. However, when the lesion does not meet the curability criteria, which is referred to as non-curative resection (or eCuraC-2 in the Japanese guidelines for gastric cancer), using additional treatment because of the possibility of LNM is the standard protocol [18,19,20,21,22,23]. The LNM rates in such lesions are relatively low (e.g., approximately 8% in EGCs) [24]. Furthermore, with the increase in the aging population, a two-fold increase in the number of new cancer cases among adults aged ≥ 65 years is expected worldwide [25]. Thus, additional treatment for all patients for SESCC or EGC with non-curative ER may be overtreatment. To date, there have been no reviews that compare the management of SESCC with EGC following non-curative ER. Hence, in this review, we describe the current knowledge and future perspectives in this field.

## 2. Non-Curative ER for SESCC

### 2.1. Non-Curative ER in the Guidelines

**Figure 1:**
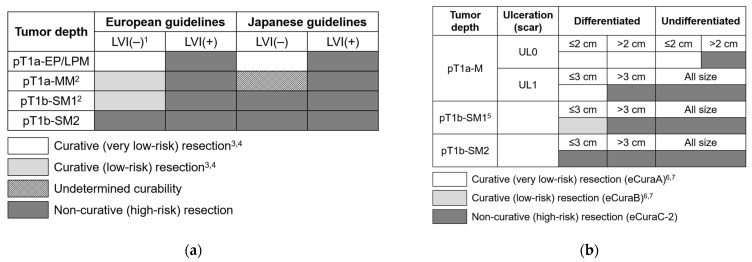
(**a**) Curability criteria after ER for SESCC; (**b**) EGC. ^1^ Poorly differentiated tumor is regarded as non-curative ER. ^2^ In the European guidelines, pT1a-MM/pT1b-SM1 with negative LVI is considered curative, but additional radiotherapy or CRT may be considered in a multidisciplinary discussion, particularly if the tumor diameter is > 20 mm. ^3^ Confined by negative horizontal and vertical margins with negative LVI. ^4^ Piecemeal resection or resection en bloc with a positive horizontal margin is regarded as non-curative ER. ^5^ A lesion with a submucosal undifferentiated component is regarded as non-curative ER (eCuraC-2) in the Japanese guidelines. ^6^ Confined by negative horizontal and vertical margins with negative LVI. ^7^ Piecemeal resection or resection en bloc with a positive horizontal margin is regarded as non-curative ER (eCuraC-1 in the Japanese guidelines). CRT, chemoradiotherapy; EGC, early gastric cancer; ER, endoscopic resection; LVI, lymphovascular invasion; pT1a-EP/LPM, tumor invasion limited to the epithelium or lamina propria mucosa; pT1a-M, intramucosal adenocarcinoma; pT1a-MM, tumor invasion confined to the muscularis mucosa; pT1b-SM1 (EGC), submucosal adenocarcinoma confined to <500 µm of the submucosa; pT1b-SM1 (SESCC), tumor invasion confined to the submucosa ≤200 µm; pT1b-SM2 (EGC), submucosal adenocarcinoma invading ≥500 µm of the submucosa; pT1b-SM2 (SESCC), tumor invasion into the submucosa >200 µm; SESCC, superficial esophageal squamous cell carcinoma.

In both European and Japanese guidelines [18,19,20,22], en bloc R0 resection for tumor invasion limited to the epithelium or lamina propria mucosa (pT1a-EP/LPM), well to moderately differentiated, and negative lymphovascular invasion (LVI) is regarded as curative (Figure 1a). Although a poorly differentiated tumor is believed to not meet the curability criteria according to the European guidelines [18], based on two reports [26,27], the Japanese guidelines do not describe differentiation [19,20,22]. When the lesion does not meet the curability criteria, the resection is considered non-curative ER, and further treatment (esophagectomy, chemoradiotherapy [CRT], or radiotherapy) is generally recommended. However, no definite recommendation has been made in the Japanese guidelines for tumor invasion confined to the muscularis mucosa (pT1a-MM) with negative LVI because of the risk of LNM [19,20,22]. According to European guidelines [18], pT1a-MM tumor invasion confined to the submucosa ≤ 200 µm (pT1b-SM1) with negative LVI is considered curative; however, additional radiotherapy or CRT may be considered in a multidisciplinary discussion, particularly if the tumor diameter is >20 mm. In this study, pT1a-MM/pT1b-SM1 with negative LVI was also regarded as a non-curative ER because of a certain LNM risk in this category.

### 2.2. LNM and Metastatic Recurrence in Non-Curative ER

Many retrospective studies on non-curative ER for SESCC have been reported. According to the largest study to date, only 34.9% of patients with non-curative ER for ESCC underwent additional treatment [28]. However, when the categories with an indefinite treatment strategy after non-curative ER, i.e., pT1a-MM/pT1b-SM1 with negative LVI, were excluded, 67.2% of patients underwent additional treatment [28]. In studies on upfront esophagectomy [29,30,31], the LNM rates in pT1a-EP/LPM were 0.0–5.6%, but the rates increased to 8–18% in pT1a-MM, 11.0–53.1% in pT1a-SM1, and 30.0–53.9% in tumor invasion into the submucosa >200 µm (pT1b-SM2). When lymphatic invasion was negative, the LNM rates were 10.3% and 28.6% for pT1a-MM and pT1b-SM1, respectively [30]. However, caution is required when applying these results while making a decision after non-curative ER because the recommended tissue slice preparation differs between surgically and endoscopically resected specimens [20,32]. To resolve this issue, calculation of the LNM rate following ER is desirable; however, unlike those with EGC, many patients with SESCC undergo CRT following non-curative ER, making it difficult to evaluate the LNM rate. Some patients do not undergo additional treatment following non-curative ER; thus, metastatic recurrence can be evaluated in pathology as a surrogate of LNM. Previous reports on pT1a-MM with negative LVI diagnosed by an endoscopically resected specimen showed that the metastatic recurrence rates were 0.0–4.3% [32,33,34], which is different from the results of esophagectomy [30]. A recent multicenter study on pT1a-MM/tumor invasion into the submucosa (pT1b-SM) diagnosed using an endoscopically resected specimen revealed that the 5-year metastatic recurrence rates in pT1a-MM and pT1b-SM1 with negative LVI and vertical margin (VM) were 2.6% and 4.3%, respectively, whereas the rate was 23.6% in the other categories (pT1b-SM2, positive LVI, or positive VM) [28]. Furthermore, unlike EGC, some patients can be curatively treated when metastatic recurrence occurs following no additional treatment for non-curative ER. A multicenter study clarified that locoregional recurrence was detected in 65.2% of patients with metastatic recurrence, and 83.3% of patients who underwent salvage treatment had no further recurrence [28]. In total, 47.8% of patients with metastatic recurrence achieved a long-term prognosis without further recurrence after salvage treatment (Table 1). This result is more favorable than that for EGCs; only 3.7% of patients with metastatic recurrence after non-curative ER without additional treatment for EGCs achieved a long-term prognosis [35,36]. Since the follow-up methods did not differ between the two studies (i.e., esophagogastroduodenoscopy and computed tomography (CT) every 6 months as much as possible), the difference may have been due to the intrinsic nature of SESCC and EGC.

**Figure 2:**
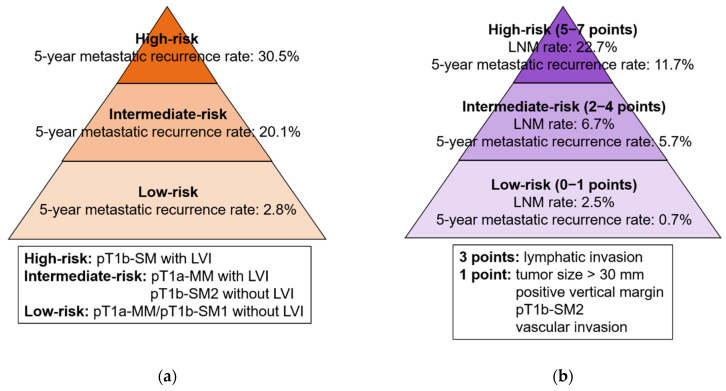
(**a**) Risk classification for LNM and/or metastatic recurrence in patients with non-curative ER for SESCC; (**b**) or EGC. EGC, early gastric cancer; ER, endoscopic resection; LNM, lymph node metastasis; LVI, lymphovascular invasion; pT1a-MM, tumor invasion confined to the muscularis mucosa; pT1b-SM, tumor invasion into the submucosa; pT1b-SM1 (SESCC), tumor invasion confined to submucosa ≤ 200 µm; pT1b-SM2 (EGC), submucosal adenocarcinoma invading ≥ 500 µm of the submucosa; pT1b-SM2 (SESCC), tumor invasion into the submucosa > 200 µm; SESCC, superficial esophageal squamous cell carcinoma.

Only one retrospective study has evaluated the risk factors for metastatic recurrence after non-curative ER without additional treatment for SESCC [28]. In this study, lymphatic invasion had the highest risk of metastatic recurrence, and pT1b-SM2 and positive VM were at significant risk of metastatic recurrence. Furthermore, risk classification for metastatic recurrence following non-curative ER without additional treatment by combining tumor depth and LVI was suggested, which is as follows: low-risk, pT1a-MM/pT1b-SM1 with negative LVI; intermediate-risk, pT1a-MM with positive LVI or pT1b-SM2 with negative LVI; and high-risk, pT1b-SM with positive LVI (Figure 2a). The 5-year metastatic recurrence rates in the low-, intermediate-, and high-risk categories were 2.8%, 20.1%, and 30.5%, respectively. Thus, this classification may reflect the risk of metastatic recurrence after non-curative ER without additional treatment; however, further validation of this classification is required.

### 2.3. Esophagectomy or CRT, the Preferable Optimal Treatment Option as an Additional Treatment following Non-Curative ER for SESCC

Esophagectomy and CRT are two recommended treatment methods for young and fit patients with non-curative ER for SESCC, but the selection of the treatment method depends on the institution [28,37,38,39,40,41]. Several studies have compared the outcomes between esophagectomy and CRT following non-curative ER, and, in most studies, recurrence was higher with additional CRT than with additional esophagectomy (3.8–27.2% vs. 0.0–11.1%; Table 2) [37,38,39,40,41,42]. These results suggest the superior effect of esophagectomy in preventing recurrence after non-curative ER for SESCC. However, high invasiveness of esophagectomy may be problematic at times. Indeed, three of the six studies had patients with treatment-related mortality during esophagectomy (1.8–7.1%), whereas all six studies had no mortality associated with CRT [37,38,39,40,41] (Table 2). Furthermore, esophagectomy may impair the quality of life (QoL) more than CRT. However, most studies had the major limitation of being unadjusted for the background of patients in the two treatment arms. To overcome this issue, a phase III, multicenter, randomized controlled trial comparing additional esophagectomy with definitive CRT for patients with clinical T1N0M0 and pT1b-SM ESCC after ESD is currently being performed in China [43]. The results of this study may clarify the optimal treatment method for SESCC following non-curative ER.

### 2.4. A Novel Treatment Method following Non-Curative ER

Two major issues in CRT are the high rate of local failure (19–31% of cases) and adverse events associated with dose escalation [44,45,46,47]. Therefore, ER and selective CRT may be minimally invasive treatment options for SESCC with a possible risk of LNM. Recently, the efficacy of ER and selective CRT for stage I ESCC has been prospectively demonstrated [48]. Although this was a single-arm confirmative trial, a favorable 3-year overall survival (OS; 92.6%) was achieved. In this trial, which included patients with clinical T1b-SM, the following protocol was determined after ER: (1) no additional treatment for pT1a-EP/LPM/MM with negative resection margins; (2) prophylactic CRT (41.4 Gy for regional lymph nodes) for pT1b-SM ESCC with negative resection margins or pT1a-EP/LPM/MM with LVI; and (3) definitive CRT (50.4 Gy with a 9 Gy boost for the primary tumor) for positive resection margins or uncollectible or uncertain margins for determining cancer-free status. In this trial, only one patient developed grade 4 cardiac ischemia according to the Common Terminology Criteria for Adverse Events, and none of the patients died from adverse events. Therefore, the safety and efficacy of this method are clinically acceptable. However, it should be noted that death from adverse events, even with prophylactic CRT following non-curative ER, has been reported [49].

### 2.5. Prognosis and Prognostic Factors

Many patients with non-curative ER for SESCC die of non-ESCC-related causes [28]. Several retrospective studies have reported the prognostic factors in patients with ER for SESCC [49,50,51,52,53,54] (Table 3), but the study populations and significant factors, except the Charlson comorbidity index (CCI), which is a 19-comorbidity tool with weighted points [55], differed across studies. Only one study evaluated the prognostic factors in patients with non-curative ER for SESCC [54]. In the study, age ≥ 75 years, male sex, CCI, prognostic nutrition index <45, as well as pathological intermediate- and high-risk categories shown in Figure 2a, were prognostic factors. Pathological factors are associated with ESCC-specific mortality, whereas other factors are mainly associated with non-ESCC-related mortality. Thus, the combined assessment of ESCC- and non-ESCC-related mortality is required for deciding on treatment strategy after non-curative ER. To date, no prospective studies evaluating the prognostic factors of ESCC in patients with ER have been reported. It is difficult to evaluate several findings, such as psychological status and cognition, in retrospective studies; thus, a prospective study investigating various tools is required.

## 3. Non-Curative ER for EGCs

### 3.1. Non-Curative ER in the Guidelines

According to European and Japanese guidelines [18,21,23], the curability criteria after ER for EGCs are en bloc R0 resection and no LVI with the following criteria: (1) non-ulcerated differentiated-type intramucosal adenocarcinoma (pT1a-M); (2) ulcerated differentiated-type pT1a-M ≤ 30 mm; (3) differentiated-type, submucosal adenocarcinoma confined to < 500 µm of the submucosa (pT1b-SM1) ≤ 30 mm; and (4) non-ulcerated undifferentiated-type pT1a-M ≤ 20 mm (Figure 1b). Lesions that do not meet these criteria are diagnosed as non-curative ER. According to the Japanese guidelines [21,23], a lesion with a submucosal undifferentiated component is regarded as non-curative because this category has been reported to be at high risk for LNM [56,57].

### 3.2. LNM in Non-Curative ER

Many studies in this field are retrospective [58]. Additional gastrectomy is the standard treatment method for non-curative ER for EGCs according to the guidelines [18,21,23]; however, approximately half of the patients underwent additional gastrectomy in the real world [35,59]. Furthermore, only approximately 20% of patients aged ≥ 80 years underwent this treatment method after non-curative ER for EGC [60]. A recent systematic review found that the LNM rate following non-curative ER was 8.1% (7.3–9.0%); however, most reports were from Korea and Japan [24]. According to a prospective study from Germany, LNM was found in 8.3% (1/12) of patients with non-curative ER [61]. Although the no-touch isolation concept is sometimes discussed to prevent the spread of cancer cells [62,63], submucosal manipulation during gastric ER does not enhance the risk of LNM [64].

Regarding risk factors for LNM in non-curative ER, a systematic review revealed that lymphatic invasion or LVI is the highest risk for LNM [24]. Furthermore, tumor size > 30 mm, positive VM, submucosal adenocarcinoma with invasion ≥ 500 µm (pT1b-SM2), and vascular invasion were risk factors for LNM. Recently, a multicenter retrospective study established a scoring system, referred to as the eCura system, to stratify the risk of LNM in a large cohort. This system consists of 5 pathological factors (3 points for lymphatic invasion; 1 point each for tumor size > 30 mm, positive VM, vascular invasion, and pT1b-SM2) with the following 3 risk categories: low-risk (2.5% LNM risk), intermediate-risk (6.7% risk), and high-risk (22.7% risk; Figure 2b) [65]. Free mobile applications are now available in English, Chinese, and Japanese [66,67]. Although this system has been internally validated [65], external validation is required in the future.

In the eCura system, 0 points are assigned to the undifferentiated type [65], even though undifferentiated-type EGCs are at a higher risk for LNM according to studies on gastrectomy [68,69]. Furthermore, a systematic review showed that this factor was not significantly associated with LNM following non-curative ER for EGCs [24]. The indication of ER for undifferentiated-type EGCs is limited (only for non-ulcerated pT1a-M ≤ 20 mm); thus, many patients with undifferentiated-type EGCs undergo gastrectomy as initial treatment. This selection bias is called the “indication issue” [70]. Since the eCura system was established in patients who underwent additional gastrectomy following non-curative ER for EGCs, caution is required when interpreting the risk of the undifferentiated type in this system. In particular, undifferentiated components in the submucosa should be noted because a high risk of LNM in this factor was demonstrated in the analysis of additional gastrectomy following non-curative ER [57]. On the other hand, the eCura system may be applicable for cases with undifferentiated-type EGCs that meet the indication criteria of ER preoperatively but result in non-curative ER because the eCura system was established based on the analysis of such lesions. One of the limitations of this system is the small number of cases of the undifferentiated type in the development cohort (150 cases) [65]; thus, it is necessary to confirm the validity of the eCura system for undifferentiated-type EGC by using a large cohort in the future.

### 3.3. Metastatic Recurrence after Non-Curative ER without Additional Treatment

The eCura system also predicts metastatic recurrence rate in patients without additional gastrectomy following non-curative ER of 0.7%, 5.7%, and 11.7% in the low-, intermediate-, and high-risk categories, respectively [71]. The very low rate of metastatic recurrence in the low-risk category may encourage clinicians to select no additional treatment following non-curative ER. However, it should be noted that the prognosis in most patients with metastatic recurrence after non-curative ER for EGC is poor [35,36] (Table 1), which differs from the results in patients with metastatic recurrence after non-curative ER for SESCC [28]. Thus, even in the low-risk category, clinicians should carefully explain this fact to the patients before selecting no additional treatment in patients with non-curative ER for EGCs. Furthermore, the timing of metastatic recurrence may differ depending on the pathology. A previous report found that lymphatic invasion was mainly related to early metastatic recurrence (≤2 years after ER), whereas vascular invasion was a risk factor only for late metastatic recurrence (>2 years after ER) in patients without additional treatment after non-curative ER for EGCs [72]. These findings may contribute to deciding the treatment strategy after non-curative ER in patients with a relatively short life expectancy.

### 3.4. Metastatic Recurrence after Additional Gastrectomy

Metastatic recurrence develops in 1.3% of patients 5 years after additional gastrectomy [73]. However, the criteria for further treatment have not been determined in the Japanese guidelines [21] because no clinical research investigating the effect of adjuvant chemotherapy has been performed in such patients. The criteria for adjuvant chemotherapy were also not determined in cases of upfront gastrectomy for EGCs in the Japanese guidelines [21], although the National Comprehensive Cancer Network guidelines recommend adjuvant chemotherapy for any T stage accompanied by positive LNM [74]. Since the prevalence of regional LNMs is at high risk for metastatic recurrence after gastrectomy [75,76,77], some retrospective studies have investigated the beneficial effect of adjuvant chemotherapy for pT1N1 gastric cancers [78,79]; however, these studies did not show any beneficial effect on tumor recurrence. A database study showed the benefit of adjuvant chemotherapy for stage IB gastric cancer patients in a competing risk analysis [80]. As such, previous reports have shown conflicting results in pT1N1 patients; thus, it is necessary to clarify further subgroups that might benefit from adjuvant chemotherapy.

Regarding risk factors for metastatic recurrence after additional gastrectomy, a recent multicenter retrospective study revealed that the presence of regional LNMs was the most important risk factor, and vascular invasion in ESD specimens was also a risk factor [72]. This study conducted a combined risk assessment of metastatic recurrence by regional LNM and vascular invasion, which exhibited a low-risk (0.0–5.6%) of recurrence during 5 years in N0 or N1 without vascular invasion and a high-risk (19.4–42.9%) in N1 with vascular invasion [72]. Although it remains unclear whether adjuvant chemotherapy can improve recurrence or prognosis in patients with additional gastrectomy, such high-risk patients may be candidates for adjuvant chemotherapy when a clinical trial is conducted.

### 3.5. Prognosis and Prognostic Factors

As with non-curative ER for SESCC, all reported studies on the prognosis of non-curative ER for EGC have been retrospective. The 5-year OS and disease-specific survival rates in patients with additional treatment after non-curative ER for EGC were 85.0–96.0% and 98.7–100%, respectively, while those in patients without additional treatment were 72.0–85.0% and 92.6–97.5%, respectively [35,81,82,83,84,85,86]. These prognoses did not differ among hospitals with different volumes [87]. These data suggest that most patients with non-curative ER for EGC died of non-gastric cancer-related causes, regardless of the treatment strategy after non-curative ER, and the difference in OS between additional and no additional treatment after non-curative ER may be largely due to the background characteristics of the patients. Many retrospective studies have investigated prognostic factors in patients with ER for EGC, and several prognostic indices, such as the American Society of Anesthesiologists’ physical status [88,89], prognostic nutrition index [90], and CCI [55], have been reported as being useful prognostic tools [91,92,93,94,95,96,97,98,99] (Table 4). However, several issues have been raised regarding the interpretation of these results. First, the study population was heterogeneous, and the results were not consistent. Second, these studies only evaluated the retrospectively available prognostic indices. Third, only one study evaluated the prognostic factors in patients with non-curative ER for EGC [94]. To overcome these issues, a large-scale prospective study in patients with non-curative ER for EGC is required.

## 4. Current Issues and Future Perspective

**Figure 3:**
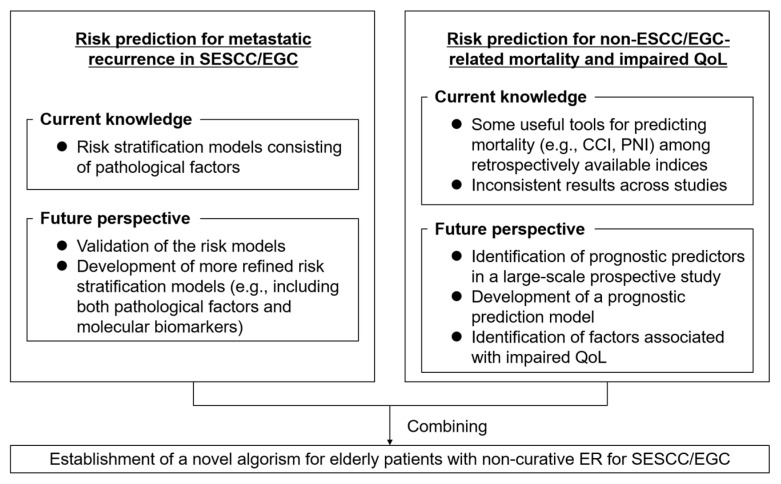
Current knowledge and future perspective for establishing a novel algorithm for older patients with non-curative ER for SESCC or EGC. CCI, Charlson comorbidity index; EGC, early gastric cancer; ER, endoscopic resection; ESCC, esophageal squamous cell carcinoma; PNI, prognostic nutrition index; QoL, quality of life; SESCC, superficial esophageal squamous cell carcinoma.

To date, significant evidence has been accumulated regarding the management of patients with non-curative ER for SESCC or EGC. However, some issues remain unresolved (Figure 3). First, although risk stratification for LNM or metastatic recurrence, after non-curative ER, by pathological factors has been developed in both SESCC and EGC [28,65], its discrimination is not high enough. For example, the areas under the curve (AUCs) for discriminating LNM and cancer-specific mortality risk after non-curative ER for EGC by the eCura system were 0.74 and 0.78, respectively [65], indicating fairly good discriminative ability. A recent study on T1 colorectal cancer established a risk stratification model for diagnosing LNM that combines microRNAs, messenger RNA, and pathological risk factors [100]. This model showed a high discriminative ability for LNM, with an AUC of 0.90. Although a risk stratification model with fewer factors may be required for easy clinical application, risk assessment with molecular biomarkers to improve the discriminative ability of LNM or metastatic recurrence risk is needed in cases with non-curative ER for SESCC or EGC.

Second, no appropriate guidelines have been established for the management of older patients with such cancers. The number of older patients with cancer is expected to increase in the next two decades worldwide [25], and the age peak of patients with ESCC or gastric cancer has already risen in Japan [101]. The recommendation for non-curative ER in the current guidelines is oncologically appropriate; however, in older patients, non-cancer-related mortality, QoL, and cancer-specific mortality are more important [102]. QoL is known as a key secondary outcome criterion, particularly when treatment is not expected to alter the patients’ OS [103]. Since older patients have a variety of physical conditions, comorbidities, etc. [104], a novel algorithm for managing older patients with SESCC or EGC should be established. The results of a currently ongoing multicenter prospective study to establish the algorithm may overcome this issue.

Third, although staging prior to ER is important for reducing non-curative ER, diagnostic performance for preoperative staging is still not satisfactory in both SESCC and EGC. In SESCC, non-magnifying and magnifying endoscopy, endoscopic ultrasonography (EUS), and CT are often used for preoperative staging. A systematic review showed better performance for diagnosing invasion depth of SESCC in EUS and magnifying endoscopy than in non-magnifying endoscopy [105]. However, most studies included in this systematic review were retrospective, which may have led to a bias in patient selection and analysis processes. In a recent prospective confirmatory trial, the addition of EUS was associated with a 6.6% increase in the proportion of overdiagnosis and a 4.5% decrease in the proportion of underdiagnosis, which indicates no improvement in the diagnostic accuracy of cancer invasion depth [106]. Thus, the routine use of EUS is now regarded as not beneficial for patients with SESCC. In EGC, non-magnifying endoscopy, EUS, and CT are used for preoperative staging. However, the diagnostic ability of CT for LNM is not sufficient. Indeed, preoperative CT could not detect LNM in 90% of patients with LNM who underwent additional gastrectomy after non-curative ER [84]. Non-extension sign is considered a reliable finding for pT1b-SM2 by non-magnifying endoscopy, but its diagnostic accuracy in a prospective e-learning study was 80% at most [107]. The efficacy of EUS for diagnosing invasion depth of EGC is controversial [108,109]; however, these are retrospective studies, and a prospective study is required to accurately identify its diagnostic utility. Recently, the usefulness of artificial intelligence for preoperative staging of gastric cancer has been reported. According to a report from China [110], the convolutional neural network (CNN) outperformed endoscopists and expert endoscopists in predicting the invasion depth of gastric cancer. A prospective comparison of the CNN with endoscopists will give further knowledge of its diagnostic ability.

## 5. Conclusions

Recent studies have found pathological risk stratifications for metastatic recurrence after non-curative ER for both SESCC and EGC, different recurrence patterns after non-curative ER between SESCC and EGC, and a novel treatment strategy for clinical T1b ESCC. These findings may help clinicians decide the treatment strategy following non-curative ER; however, some issues remain to be resolved for optimal therapeutic decision-making in such patients. Considering the aging of society in the near future, a novel algorithm for deciding the treatment strategy in older patients with non-curative ER for SESCC or EGC is needed.

## Figures and Tables

**Table 1:** Comparison between SESCC and EGC cases with metastatic recurrence after non-curative ER without additional treatment.

	SESCC	EGC
The rate of detection as locoregional recurrence among patients with metastatic recurrence	65.2%	21.4%
The rate of no further recurrence among patients undergoing salvage treatment for metastatic recurrence	83.3%	20.0%
The rate of patients with long-term survival and no further recurrence after salvage treatment among patients with metastatic recurrence	47.8%	3.7%

EGC, early gastric cancer; ER, endoscopic resection; SESCC, superficial esophageal squamous cell carcinoma.

**Table 2:** Reports comparing additional esophagectomy with CRT after non-curative ER for SESCC.

Authors, Year	No. of Cases		Recurrence	Treatment-Related Mortality
	Additional Esophagectomy	Additional CRT		
Ikeda et al., 2015 [37]	15	11	0 (0.0%) vs. 3 (27.2%)	1 (6.6%) vs. 0 (0.0%)
Koterazawa et al., 2018 [38]	28	31	0 (0.0%) vs. 5 (16.1%)	2 (7.1%) vs. 0 (0.0%)
Suzuki et al., 2018 [39]	16	16	0 (0.0%) vs. 1 (6.3%)	0 (0.0%) vs. 0 (0.0%)
Kanie et al., 2021 [40]	56	52	0 (0.0%) vs. 2 (3.8%)	1 (1.8%) vs. 0 (0.0%)
Miyata et al., 2021 [41]	37	123	2 (5.4%) vs. 16 (13.0%)	0 (0.0%) vs. (0.0%)
Kadota et al., 2022 [42]	18	50	2 (11.1%) vs. 2 (4.0%)	0 (0.0%) vs. (0.0%)

CRT, chemoradiotherapy; ER, endoscopic resection; SESCC, superficial esophageal squamous cell carcinoma.

**Table 3:** Reports on prognostic factors in patients with ER for SESCC.

Authors, Year	Study Population	No. of Subjects	Study Design	Prognostic Factors
Nakajo et al., 2019 [50]	75 years	360	Multicenter, retrospective	CCI ≥ 2
Ogata et al., 2021 [49]	All	407	Single-center, retrospective	Early mortality: ECOG-PS ≥ 2, CCI ≥ 2; Late mortality: ECOG-PS ≥ 2, CCI ≥ 2, age ≥ 80 years
Suzuki et al., 2021 [51]	pT1a-EP/LPM/MM or pT1b-SM1	286	Single-center, retrospective	PNI < 45, CCI ≥ 3
Iwai et al., 2021 [52]	All	659	Multicenter, retrospective	pT1a-MM/pT1b-SM1, pT1b-SM2, CCI ≥ 3, PNI ≤ 47.75
Hirano et al., 2022 [53]	PS-matched cohort	138	Single-center, retrospective	ASA-PS = 3
Shimada et al., 2022 [54]	pT1a-MM/pT1b-SM	593	Multicenter, retrospective	Male, CCI ≥ 3, ≥ 75 years, PNI < 45, pathological intermediate-/high-risk ^1^

^1^ According to the pathological risk classification after non-curative ER for SESCC [28]. ASA-PS, American Society of Anesthesiologists physical status; CCI, Charlson comorbidity index; ECOG-PS, Eastern Cooperative Oncology Group performance status; ER, endoscopic resection; PNI, prognostic nutrition index; PS, propensity score; pT1a-EP/LPM, tumor invasion confined to the epithelium or lamina propria mucosa; pT1a-MM, tumor invasion confined to the muscularis mucosa; pT1b-SM1, tumor invasion confined to the submucosa ≤200 µm; pT1b-SM2, tumor invasion into the submucosa >200 µm; SESCC, superficial esophageal squamous cell carcinoma.

**Table 4:** Reports on prognostic factors in patients with ER for EGC.

Authors, Year	Study Population	No. of Subjects	Study Design	Prognostic Factors
Yoshifuku et al., 2016 [91]	≥85 years	85	Single-center, retrospective	ASA-PS ≥ 2
Sekiguchi et al., 2017 [92]	≥85 years	108	Single-center, retrospective	PNI < 44.6
Iwai et al., 2018 [93]	All	585	Single-center, retrospective	CCI ≥ 3, ECOG-PS ≥ 2, PNI < 47.7
Toya et al., 2019 [94]	≥75 years, non-curative ER	87	Single-center, retrospective	CCI ≥ 3
Tanoue et al., 2019 [95]	PS-matched cohort	178	Single-center, retrospective	ASA-PS = 3.
Ogata et al., 2022 [96]	All (including surgery)	1439	Single-center, retrospective	Early mortality: age ≥ 85 years, CCI ≥ 2, ASA-PS ≥ 3, ECOG-PS ≥ 2, CAR ≥ 0.028, eCuraC-2-intermediate/high ^1^, low PMI; Late mortality: age ≥ 75 years, CCI ≥ 2, ASA-PS ≥ 3, ECOG-PS ≥ 2, CAR ≥ 0.028
Miyahara et al., 2022 [97]	≥80 years (including surgery)	535	Single-center, retrospective	age > 80 years, male, ECOG-PS ≥ 2, CCI ≥ 2, BMI ≤ 21.875, PNI ≤ 46.7
Waki et al., 2022 [98]	≥75 years	400	Single-center, retrospective	ECOG-PS ≥ 2, PNI < 49.1, eCuraC-2
Toya et al., 2022 [99]	≥85 years	740	Multicenter, retrospective	GNRI, CCI

^1^ Pathological risk classification among patients with non-curative ER (eCuraC-2) was based on the eCura system [65]. ASA-PS, American Society of Anesthesiologists physical status; BMI, body mass index; CAR, C-reactive protein/albumin ratio; CCI, Charlson comorbidity index; ECOG-PS, Eastern Cooperative Oncology Group performance status; EGC, early gastric cancer; ER, endoscopic resection; GNRI, Geriatric Nutritional Risk Index; PMI, psoas muscle mass index; PNI, prognostic nutrition index; PS, propensity score.

## References

[1] Muto M., Minashi K., Yano T., Saito Y., Oda I., Nonaka S., Omori T., Sugiura H., Goda K., Kaise M. (2010). Early detection of superficial squamous cell carcinoma in the head and neck region and esophagus by narrow band imaging: A multicenter randomized controlled trial. J. Clin. Oncol..

[2] Hatta W., Koike T., Ogata Y., Kondo Y., Ara N., Uno K., Asano N., Imatani A., Masamune A. (2021). Comparison of magnifying endoscopy with blue light imaging and narrow band imaging for determining the invasion depth of superficial esophageal squamous cell carcinoma by the Japanese Esophageal Society’s intrapapillary capillary loop classification. Diagnostics.

[3] Ono S., Kawada K., Dohi O., Kitamura S., Koike T., Hori S., Kanzaki H., Murao T., Yagi N., Sasaki F. (2021). Linked color imaging focused on neoplasm detection in the upper gastrointestinal tract: A randomized trial. Ann. Intern. Med..

[4] Yoshida N., Doyama H., Yano T., Horimatsu T., Uedo N., Yamamoto Y., Kakushima N., Kanzaki H., Hori S., Yao K. (2021). Early gastric cancer detection in high-risk patients: A multicentre randomised controlled trial on the effect of second-generation narrow band imaging. Gut.

[5] Katada C., Yokoyama T., Yano T., Kaneko K., Oda I., Shimizu Y., Doyama H., Koike T., Takizawa K., Hirao M. (2016). Alcohol consumption and multiple dysplastic lesions increase risk of squamous cell carcinoma in the esophagus, head, and neck. Gastroenterology.

[6] Pimentel-Nunes P., Dinis-Ribeiro M., Ponchon T., Repici A., Vieth M., De Ceglie A., Amato A., Berr F., Bhandari P., Bialek A. (2015). Endoscopic submucosal dissection: European Society of Gastrointestinal Endoscopy (ESGE) Guideline. Endoscopy.

[7] Hatta W., Gotoda T., Koike T., Masamune A. (2020). History and future perspectives in Japanese guidelines for endoscopic resection of early gastric cancer. Dig. Endosc..

[8] Probst A., Aust D., Markl B., Anthuber M., Messmann H. (2015). Early esophageal cancer in Europe: Endoscopic treatment by endoscopic submucosal dissection. Endoscopy.

[9] Tsujii Y., Nishida T., Nishiyama O., Yamamoto K., Kawai N., Yamaguchi S., Yamada T., Yoshio T., Kitamura S., Nakamura T. (2015). Clinical outcomes of endoscopic submucosal dissection for superficial esophageal neoplasms: A multicenter retrospective cohort study. Endoscopy.

[10] Tanabe S., Ishido K., Matsumoto T., Kosaka T., Oda I., Suzuki H., Fujisaki J., Ono H., Kawata N., Oyama T. (2017). Long-term outcomes of endoscopic submucosal dissection for early gastric cancer: A multicenter collaborative study. Gastric Cancer.

[11] Park H.C., Kim D.H., Gong E.J., Na H.K., Ahn J.Y., Lee J.H., Jung K.W., Choi K.D., Song H.J., Lee G.H. (2016). Ten-year experience of esophageal endoscopic submucosal dissection of superficial esophageal neoplasms in a single center. Korean J. Intern. Med..

[12] Xu J.Q., Zhang Z.C., Chen W.F., Xu M.D., Chen S.Y., Zhong Y.S., Zhang Y.Q., Hu J.W., Cai M.Y., Yao L.Q. (2022). Repeat endoscopic submucosal dissection as salvage treatment for local recurrence of esophageal squamous cell carcinoma after initial endoscopic submucosal dissection. Gastrointest. Endosc..

[13] Kim G.H., Choi K.D., Ko Y., Park T., Kim K.W., Park S.Y., Na H.K., Ahn J.Y., Lee J.H., Jung K.W. (2021). Impact of comorbidities, sarcopenia, and nutritional status on the long-term outcomes after endoscopic submucosal dissection for early gastric cancer in elderly patients aged ≥ 80 years. Cancers.

[14] Zhang H.H., Soyfoo M.D., Cao J.L., Sang H.M., Xu S.F., Jiang J.X. (2022). Histopathological characteristics and therapeutic outcomes of endoscopic submucosal dissection for gastric high-grade intraepithelial neoplasia. J. Laparoendosc. Adv. Surg. Tech. A.

[15] Hatta W., Koike T., Abe H., Ogata Y., Saito M., Jin X., Kanno T., Uno K., Asano N., Imatani A. (2021). Recent approach for preventing complications in upper gastrointestinal endoscopic submucosal dissection. DEN Open.

[16] Berger A., Rahmi G., Perrod G., Pioche M., Canard J.M., Cesbron-Metivier E., Boursier J., Samaha E., Vienne A., Lepilliez V. (2019). Long-term follow-up after endoscopic resection for superficial esophageal squamous cell carcinoma: A multicenter Western study. Endoscopy.

[17] Fleischmann C., Probst A., Ebigbo A., Faiss S., Schumacher B., Allgaier H.P., Dumoulin F.L., Steinbrueck I., Anzinger M., Marienhagen J. (2021). Endoscopic submucosal dissection in Europe: Results of 1000 neoplastic lesions from the German endoscopic submucosal dissection registry. Gastroenterology.

[18] Pimentel-Nunes P., Libanio D., Bastiaansen B.A.J., Bhandari P., Bisschops R., Bourke M.J., Esposito G., Lemmers A., Maselli R., Messmann H. (2022). Endoscopic submucosal dissection for superficial gastrointestinal lesions: European Society of Gastrointestinal Endoscopy (ESGE) Guideline-Update 2022. Endoscopy.

[19] Kitagawa Y., Uno T., Oyama T., Kato K., Kato H., Kawakubo H., Kawamura O., Kusano M., Kuwano H., Takeuchi H. (2019). Esophageal cancer practice guidelines 2017 edited by the Japan Esophageal Society: Part 1. Esophagus.

[20] Kitagawa Y., Uno T., Oyama T., Kato K., Kato H., Kawakubo H., Kawamura O., Kusano M., Kuwano H., Takeuchi H. (2019). Esophageal cancer practice guidelines 2017 edited by the Japan esophageal society: Part 2. Esophagus.

[21] Japanese Gastric Cancer Association (2021). Japanese gastric cancer treatment guidelines 2018 (5th edition). Gastric Cancer.

[22] Ishihara R., Arima M., Iizuka T., Oyama T., Katada C., Kato M., Goda K., Goto O., Tanaka K., Yano T. (2020). Endoscopic submucosal dissection/endoscopic mucosal resection guidelines for esophageal cancer. Dig. Endosc..

[23] Ono H., Yao K., Fujishiro M., Oda I., Uedo N., Nimura S., Yahagi N., Iishi H., Oka M., Ajioka Y. (2021). Guidelines for endoscopic submucosal dissection and endoscopic mucosal resection for early gastric cancer (second edition). Dig. Endosc..

[24] Hatta W., Gotoda T., Kanno T., Yuan Y., Koike T., Moayyedi P., Masamune A. (2020). Prevalence and risk factors for lymph node metastasis after non-curative endoscopic resection for early gastric cancer: A systematic review and meta-analysis. J. Gastroenterol..

[25] Pilleron S., Sarfati D., Janssen-Heijnen M., Vignat J., Ferlay J., Bray F., Soerjomataram I. (2019). Global cancer incidence in older adults, 2012 and 2035: A population-based study. Int. J. Cancer.

[26] Xu W., Liu X.B., Li S.B., Yang Z.H., Tong Q. (2020). Prediction of lymph node metastasis in superficial esophageal squamous cell carcinoma in Asia: A systematic review and meta-analysis. Dis. Esophagus.

[27] Ye B., Zhang X., Su Y., Hao S., Teng H., Guo X., Yang Y., Sun Y., Mao T., Li Z. (2021). The possibility of endoscopic treatment of cN0 submucosal esophageal cancer: Results from a surgical cohort. Surg. Endosc..

[28] Hatta W., Koike T., Takahashi S., Shimada T., Hikichi T., Toya Y., Tanaka I., Onozato Y., Hamada K., Fukushi D. (2021). Risk of metastatic recurrence after endoscopic resection for esophageal squamous cell carcinoma invading into the muscularis mucosa or submucosa: A multicenter retrospective study. J. Gastroenterol..

[29] Tajima Y., Nakanishi Y., Tachimori Y., Kato H., Watanabe H., Yamaguchi H., Yoshimura K., Kusano M., Shimoda T. (2000). Significance of involvement by squamous cell carcinoma of the ducts of esophageal submucosal glands. Analysis of 201 surgically resected superficial squamous cell carcinomas. Cancer.

[30] Eguchi T., Nakanishi Y., Shimoda T., Iwasaki M., Igaki H., Tachimori Y., Kato H., Yamaguchi H., Saito D., Umemura S. (2006). Histopathological criteria for additional treatment after endoscopic mucosal resection for esophageal cancer: Analysis of 464 surgically resected cases. Mod. Pathol..

[31] Bollschweiler E., Baldus S.E., Schroder W., Prenzel K., Gutschow C., Schneider P.M., Holscher A.H. (2006). High rate of lymph-node metastasis in submucosal esophageal squamous-cell carcinomas and adenocarcinomas. Endoscopy.

[32] Akutsu Y., Uesato M., Shuto K., Kono T., Hoshino I., Horibe D., Sazuka T., Takeshita N., Maruyama T., Isozaki Y. (2013). The overall prevalence of metastasis in T1 esophageal squamous cell carcinoma: A retrospective analysis of 295 patients. Ann. Surg..

[33] Katada C., Muto M., Momma K., Arima M., Tajiri H., Kanamaru C., Ooyanagi H., Endo H., Michida T., Hasuike N. (2007). Clinical outcome after endoscopic mucosal resection for esophageal squamous cell carcinoma invading the muscularis mucosae—A multicenter retrospective cohort study. Endoscopy.

[34] Yamashina T., Ishihara R., Nagai K., Matsuura N., Matsui F., Ito T., Fujii M., Yamamoto S., Hanaoka N., Takeuchi Y. (2013). Long-term outcome and metastatic risk after endoscopic resection of superficial esophageal squamous cell carcinoma. Am. J. Gastroenterol..

[35] Hatta W., Gotoda T., Oyama T., Kawata N., Takahashi A., Yoshifuku Y., Hoteya S., Nakamura K., Hirano M., Esaki M. (2017). Is radical surgery necessary in all patients who do not meet the curative criteria for endoscopic submucosal dissection in early gastric cancer? A multicenter retrospective study in Japan. J. Gastroenterol..

[36] Takizawa K., Hatta W., Gotoda T., Kawata N., Nakagawa M., Takahashi A., Esaki M., Mitoro A., Yamada S., Tanaka K. (2019). Recurrence patterns and outcomes of salvage surgery in cases of non-curative endoscopic submucosal dissection without additional radical surgery for early gastric cancer. Digestion.

[37] Ikeda A., Hoshi N., Yoshizaki T., Fujishima Y., Ishida T., Morita Y., Ejima Y., Toyonaga T., Kakechi Y., Yokosaki H. (2015). Endoscopic submucosal dissection (ESD) with additional therapy for superficial esophageal cancer with submucosal invasion. Intern. Med..

[38] Koterazawa Y., Nakamura T., Oshikiri T., Kanaji S., Tanaka S., Ishida T., Yamashita K., Matsuda T., Morita Y., Suzuki S. (2018). A comparison of the clinical outcomes of esophagectomy and chemoradiotherapy after non-curative endoscopic submucosal dissection for esophageal squamous cell carcinoma. Surg. Today.

[39] Suzuki G., Yamazaki H., Aibe N., Masui K., Sasaki N., Shimizu D., Kimoto T., Shiozaki A., Dohi O., Fujiwara H. (2018). Endoscopic submucosal dissection followed by chemoradiotherapy for superficial esophageal cancer: Choice of new approach. Radiat. Oncol..

[40] Kanie Y., Okamura A., Asari T., Maruyama S., Sakamoto K., Fujiwara D., Kanamori J., Imamura Y., Ishiyama A., Yoshio T. (2021). Additional treatment following non-curative endoscopic resection for esophageal squamous cell carcinoma: A comparison of outcomes between esophagectomy and chemoradiotherapy. Ann. Surg. Oncol..

[41] Miyata H., Sugimura K., Kanemura T., Takeoka T., Yamamoto M., Shinno N., Hara H., Omori T., Yamamoto S., Ishihara R. (2021). Clinical outcome of additional esophagectomy after endoscopic treatment for superficial esophageal cancer. Ann. Surg. Oncol..

[42] Kadota T., Sato D., Inaba A., Nishihara K., Takashima K., Nakajo K., Yukami H., Mishima S., Sawada K., Kotani D. (2022). Long-term clinical outcomes of patients diagnosed with pT1a-muscularis mucosae with lymphovascular invasion or pT1b after endoscopic resection for cT1N0M0 esophageal squamous cell carcinoma. Esophagus.

[43] Yang Y., Su Y., Zhang X., Liu J., Zhang H., Li B., Hua R., Tan L., Chen H., Li Z. (2020). Esophagectomy versus definitive chemoradiotherapy for patients with clinical stage N0 and pathological stage T1b esophageal squamous cell carcinoma after endoscopic submucosal dissection: Study protocol for a multicenter randomized controlled trial (Ad-ESD Trial). Trials.

[44] Kato H., Sato A., Fukuda H., Kagami Y., Udagawa H., Togo A., Ando N., Tanaka O., Shinoda M., Yamana H. (2009). A phase II trial of chemoradiotherapy for stage I esophageal squamous cell carcinoma: Japan Clinical Oncology Group Study (JCOG9708). Jpn. J. Clin. Oncol..

[45] Kawaguchi G., Sasamoto R., Abe E., Ohta A., Sato H., Tanaka K., Maruyama K., Kaizu M., Ayukawa F., Yamana N. (2015). The effectiveness of endoscopic submucosal dissection followed by chemoradiotherapy for superficial esophageal cancer. Radiat. Oncol..

[46] Yoshimizu S., Yoshio T., Ishiyama A., Tsuchida T., Horiuchi Y., Omae M., Hirasawa T., Asari T., Chin K., Fujisaki J. (2018). Long-term outcomes of combined endoscopic resection and chemoradiotherapy for esophageal squamous cell carcinoma with submucosal invasion. Dig. Liver. Dis..

[47] Tsou Y.K., Lee C.H., Le P.H., Chen B.H. (2020). Adjuvant therapy for pT1a-m3/pT1b esophageal squamous cell carcinoma after endoscopic resection: Esophagectomy or chemoradiotherapy? A critical review. Crit. Rev. Oncol. Hematol..

[48] Minashi K., Nihei K., Mizusawa J., Takizawa K., Yano T., Ezoe Y., Tsuchida T., Ono H., Iizuka T., Hanaoka N. (2019). Efficacy of endoscopic resection and selective chemoradiotherapy for stage I esophageal squamous cell carcinoma. Gastroenterology.

[49] Ogata Y., Hatta W., Koike T., Saito M., Jin X., Nakagawa K., Kanno T., Uno K., Asano N., Imatani A. (2021). Predictors of early and late mortality after endoscopic resection for esophageal squamous cell carcinoma. Tohoku J. Exp. Med..

[50] Nakajo K., Abe S., Oda I., Ishihara R., Tanaka M., Yoshio T., Katada C., Yano T. (2019). Impact of the Charlson Comorbidity Index on the treatment strategy and survival in elderly patients after non-curative endoscopic submucosal dissection for esophageal squamous cell carcinoma: A multicenter retrospective study. J. Gastroenterol..

[51] Suzuki T., Furukawa K., Funasaka K., Ishikawa E., Sawada T., Maeda K., Yamamura T., Ishikawa T., Ohno E., Nakamura M. (2021). Long-term prognostic predictors of esophageal squamous cell carcinoma potentially indicated for endoscopic submucosal dissection. Digestion.

[52] Iwai N., Dohi O., Yamada S., Harusato A., Horie R., Yasuda T., Yamada N., Horii Y., Majima A., Zen K. (2022). Prognostic risk factors associated with esophageal squamous cell carcinoma patients undergoing endoscopic submucosal dissection: A multicenter cohort study. Surg. Endosc..

[53] Hirano S., Nagami Y., Yamamura M., Tanoue K., Sakai T., Maruyama H., Ominami M., Nadatani Y., Fukunaga S., Otani K. (2022). Evaluation of long-term survival in patients with severe comorbidities after endoscopic submucosal dissection for esophageal squamous cell carcinoma. Surg. Endosc..

[54] Shimada T., Hatta W., Takahashi S., Koike T., Ohira T., Hikichi T., Toya Y., Tanaka I., Onozato Y., Hamada K. (2022). A combined assessment of clinical and pathological prognostic factors for deciding treatment strategies for esophageal squamous cell carcinoma invading into the muscularis mucosa or submucosa after endoscopic submucosal dissection. Dig. Endosc..

[55] Charlson M.E., Pompei P., Ales K.L., MacKenzie C.R. (1987). A new method of classifying prognostic comorbidity in longitudinal studies: Development and validation. J. Chronic Dis..

[56] Jung D.H., Bae Y.S., Yoon S.O., Lee Y.C., Kim H., Noh S.H., Park H., Choi S.H., Kim J.H., Kim H. (2015). Poorly differentiated carcinoma component in submucosal layer should be considered as an additional criterion for curative endoscopic resection of early gastric cancer. Ann. Surg. Oncol..

[57] Miyahara K., Hatta W., Nakagawa M., Oyama T., Kawata N., Takahashi A., Yoshifuku Y., Hoteya S., Hirano M., Esaki M. (2018). The role of an undifferentiated component in submucosal invasion and submucosal invasion depth after endoscopic submucosal dissection for early gastric cancer. Digestion.

[58] Hatta W., Gotoda T., Koike T., Masamune A. (2020). A recent argument for the use of endoscopic submucosal dissection for early gastric cancers. Gut Liver.

[59] Suzuki H., Takizawa K., Hirasawa T., Takeuchi Y., Ishido K., Hoteya S., Yano T., Tanaka S., Endo M., Nakagawa M. (2019). Short-term outcomes of multicenter prospective cohort study of gastric endoscopic resection: ‘Real-world evidence’ in Japan. Dig. Endosc..

[60] Esaki M., Hatta W., Shimosegawa T., Oyama T., Kawata N., Takahashi A., Oka S., Hoteya S., Nakagawa M., Hirano M. (2019). Age affects clinical management after non-curative endoscopic submucosal dissection for early gastric cancer. Dig. Dis..

[61] Probst A., Schneider A., Schaller T., Anthuber M., Ebigbo A., Messmann H. (2017). Endoscopic submucosal dissection for early gastric cancer: Are expanded resection criteria safe for Western patients?. Endoscopy.

[62] Fujita J., Uyama I., Sugioka A., Komori Y., Matsui H., Hasumi A. (2001). Laparoscopic right hemicolectomy with radical lymph node dissection using the no-touch isolation technique for advanced colon cancer. Surg. Today.

[63] Gall T.M., Jacob J., Frampton A.E., Krell J., Kyriakides C., Castellano L., Stebbing J., Jiao L.R. (2014). Reduced dissemination of circulating tumor cells with no-touch isolation surgical technique in patients with pancreatic cancer. JAMA Surg..

[64] Ito H., Gotoda T., Oyama T., Kawata N., Takahashi A., Yoshifuku Y., Hoteya S., Nakagawa M., Hatta W., Hirano M. (2018). Long-term oncological outcomes of submucosal manipulation during non-curative endoscopic submucosal dissection for submucosal invasive gastric cancer: A multicenter retrospective study in Japan. Surg. Endosc..

[65] Hatta W., Gotoda T., Oyama T., Kawata N., Takahashi A., Yoshifuku Y., Hoteya S., Nakagawa M., Hirano M., Esaki M. (2017). A scoring system to stratify curability after endoscopic submucosal dissection for early gastric cancer: “eCura system”. Am. J. Gastroenterol..

[66] For iOS. https://apps.apple.com/app/ecura/id1490245005.

[67] For Android. https://play.google.com/store/apps/details?id=hatta.eCura.

[68] Gotoda T., Yanagisawa A., Sasako M., Ono H., Nakanishi Y., Shimoda T., Kato Y. (2000). Incidence of lymph node metastasis from early gastric cancer: Estimation with a large number of cases at two large centers. Gastric Cancer.

[69] Hirasawa T., Gotoda T., Miyata S., Kato Y., Shimoda T., Taniguchi H., Fujisaki J., Sano T., Yamaguchi T. (2009). Incidence of lymph node metastasis and the feasibility of endoscopic resection for undifferentiated-type early gastric cancer. Gastric Cancer.

[70] Lee H., Lee J.H. (2015). Expanding indications of endoscopic submucosal dissection for early gastric cancer: Hope or hype?. Gut Liver.

[71] Hatta W., Gotoda T., Oyama T., Kawata N., Takahashi A., Yoshifuku Y., Hoteya S., Nakagawa M., Hirano M., Esaki M. (2018). Is the eCura system useful for selecting patients who require radical surgery after non-curative endoscopic submucosal dissection for early gastric cancer? A comparative study. Gastric Cancer.

[72] Yamada S., Hatta W., Shimosegawa T., Takizawa K., Oyama T., Kawata N., Takahashi A., Oka S., Hoteya S., Nakagawa M. (2019). Different risk factors between early and late cancer recurrences in patients without additional surgery after non-curative endoscopic submucosal dissection for early gastric cancer. Gastrointest. Endosc..

[73] Hatta W., Gotoda T., Oyama T., Kawata N., Takahashi A., Oka S., Hoteya S., Nakagawa M., Hirano M., Esaki M. (2019). Is additional surgery always sufficient for preventing recurrence after endoscopic submucosal dissection with curability C-2 for early gastric cancer?. Ann. Surg. Oncol..

[74] Ajani J.A., Bentrem D.J., Besh S., D’Amico T.A., Das P., Denlinger C., Fakih M.G., Fuchs C.S., Gerdes H., Glasgow R.E. (2013). Gastric cancer, version 2.2013: Featured updates to the NCCN Guidelines. J. Natl. Compr. Cancer Netw..

[75] Lai J.F., Kim S., Kim K., Li C., Oh S.J., Hyung W.J., Rha S.Y., Chung H.C., Choi S.H., Wang L.B. (2009). Prediction of recurrence of early gastric cancer after curative resection. Ann. Surg. Oncol..

[76] Ikeda Y., Saku M., Kishihara F., Maehara Y. (2005). Effective follow-up for recurrence or a second primary cancer in patients with early gastric cancer. Br. J. Surg..

[77] Folli S., Morgagni P., Roviello F., De Manzoni G., Marrelli D., Saragoni L., Leo A.D., Gaudio M., Nanni O., Carli A. (2001). Risk factors for lymph node metastases and their prognostic significance in early gastric cancer (EGC) for the Italian Research Group for Gastric Cancer (IRGGC). Jpn. J. Clin. Oncol..

[78] Shin H.B., An J.Y., Lee S.H., Choi Y.Y., Kim J.W., Sohn S.S., Noh S.H. (2017). Is adjuvant chemotherapy necessary in pT1N1 gastric cancer?. BMC Cancer.

[79] Kim S.M., An J.Y., Lee J., Sohn T.S., Kim S. (2018). Adjuvant chemotherapy versus chemoradiotherapy versus surgery alone for early gastric cancer with one or two lymph node metastasis. Ann. Surg. Oncol..

[80] Xie Y., Du D., Song X., Li X., Ni Z., Huang H. (2022). The role of chemotherapy in patients with stage IB gastric adenocarcinoma: A real-world competing risk analysis. World J. Surg. Oncol..

[81] Kim E.R., Lee H., Min B.H., Lee J.H., Rhee P.L., Kim J.J., Kim K.M., Kim S. (2015). Effect of rescue surgery after non-curative endoscopic resection of early gastric cancer. Br. J. Surg..

[82] Yang H.J., Kim S.G., Lim J.H., Choi J., Im J.P., Kim J.S., Kim W.H., Jung H.C. (2015). Predictors of lymph node metastasis in patients with non-curative endoscopic resection of early gastric cancer. Surg. Endosc..

[83] Suzuki H., Oda I., Abe S., Sekiguchi M., Nonaka S., Yoshinaga S., Saito Y., Fukagawa T., Katai H. (2017). Clinical outcomes of early gastric cancer patients after non-curative endoscopic submucosal dissection in a large consecutive patient series. Gastric Cancer.

[84] Kawata N., Kakushima N., Takizawa K., Tanaka M., Makuuchi R., Tokunaga M., Tanizawa Y., Bando E., Kawamura T., Sugino T. (2017). Risk factors for lymph node metastasis and long-term outcomes of patients with early gastric cancer after non-curative endoscopic submucosal dissection. Surg. Endosc..

[85] Kikuchi S., Kuroda S., Nishizaki M., Kagawa T., Kanzaki H., Kawahara Y., Kagawa S., Tanaka T., Okada H., Fujiwara T. (2017). Management of early gastric cancer that meet the indication for radical lymph node dissection following endoscopic resection: A retrospective cohort analysis. BMC Surg..

[86] Yano T., Ishido K., Tanabe S., Wada T., Azuma M., Kawanishi N., Yamane S., Watanabe A., Katada C., Koizumi W. (2018). Long-term outcomes of patients with early gastric cancer found to have lesions for which endoscopic treatment is not indicated on histopathological evaluation after endoscopic submucosal dissection. Surg. Endosc..

[87] Dohi O., Hatta W., Gotoda T., Naito Y., Oyama T., Kawata N., Takahashi A., Oka S., Hoteya S., Nakagawa M. (2019). Long-term outcomes after non-curative endoscopic submucosal dissection for early gastric cancer according to hospital volumes in Japan: A multicenter propensity-matched analysis. Surg. Endosc..

[88] Oken M.M., Creech R.H., Tormey D.C., Horton J., Davis T.E., McFadden E.T., Carbone P.P. (1982). Toxicity and response criteria of the Eastern Cooperative Oncology Group. Am. J. Clin. Oncol..

[89] American Society of Anesthesiologists Task Force on Sedation and Analgesia by Non-Anesthesiologists (2002). Practice guidelines for sedation and analgesia by non-anesthesiologists. Anesthesiology.

[90] Onodera T., Goseki N., Kosaki G. (1984). Prognostic nutritional index in gastrointestinal surgery of malnourished cancer patients. Nihon Geka Gakkai Zasshi.

[91] Yoshifuku Y., Oka S., Tanaka S., Sanomura Y., Miwata T., Numata N., Hiyama T., Chayama K. (2016). Long-term prognosis after endoscopic submucosal dissection for early gastric cancer in super-elderly patients. Surg. Endosc..

[92] Sekiguchi M., Oda I., Suzuki H., Abe S., Nonaka S., Yoshinaga S., Taniguchi H., Sekine S., Saito Y. (2017). Clinical outcomes and prognostic factors in gastric cancer patients aged ≥85 years undergoing endoscopic submucosal dissection. Gastrointest. Endosc..

[93] Iwai N., Dohi O., Naito Y., Inada Y., Fukui A., Takayama S., Ogita K., Terasaki K., Nakano T., Ueda T. (2018). Impact of the Charlson comorbidity index and prognostic nutritional index on prognosis in patients with early gastric cancer after endoscopic submucosal dissection. Dig. Endosc..

[94] Toya Y., Endo M., Nakamura S., Akasaka R., Yanai S., Kawasaki K., Koeda K., Eizuka M., Fujita Y., Uesugi N. (2019). Long-term outcomes and prognostic factors with non-curative endoscopic submucosal dissection for gastric cancer in elderly patients aged ≥ 75 years. Gastric Cancer.

[95] Tanoue K., Fukunaga S., Nagami Y., Sakai T., Maruyama H., Ominami M., Otani K., Hosomi S., Tanaka F., Taira K. (2019). Long-term outcome of endoscopic submucosal dissection for early gastric cancer in patients with severe comorbidities: A comparative propensity score analysis. Gastric Cancer.

[96] Ogata Y., Hatta W., Ohara Y., Koike T., Abe H., Saito M., Jin X., Kanno T., Uno K., Asano N. (2022). Predictors of early and late mortality after the treatment for early gastric cancers. Dig. Endosc..

[97] Miyahara K., Ishida M., Kono Y., Hirata T., Obayashi Y., Gotoda T., Ninomiya Y., Moritou Y., Kunihiro M., Kubota T. (2022). Prognosis after curative resection for stage IA gastric cancer in elderly patients: Endoscopic submucosal dissection versus surgery. Surg. Today.

[98] Waki K., Shichijo S., Uedo N., Takeuchi Y., Maekawa A., Kanesaka T., Takeuchi Y., Higashino K., Ishihara R., Tanaka Y. (2022). Long-term outcomes after endoscopic resection for late-elderly patients with early gastric cancer. Gastrointest. Endosc..

[99] Toya Y., Shimada T., Hamada K., Watanabe K., Nakamura J., Fukushi D., Hatta W., Shinkai H., Ito H., Matsuhashi T. (2022). Prediction model of 3-year survival after endoscopic submucosal dissection for early gastric cancer in elderly patients aged ≥ 85 years: EGC-2 model. J. Cancer Res. Clin. Oncol..

[100] Wada Y., Shimada M., Murano T., Takamaru H., Morine Y., Ikemoto T., Saito Y., Balaguer F., Bujanda L., Pellise M. (2021). A liquid biopsy assay for noninvasive identification of lymph node metastases in T1 colorectal cancer. Gastroenterology.

[101] Hatta W., Gotoda T., Koike T., Masamune A. (2020). Management following endoscopic resection in elderly patients with early-stage upper gastrointestinal neoplasia. Dig. Endosc..

[102] Hatta W., Gotoda T., Koike T., Uno K., Asano N., Imatani A., Masamune A. (2022). Is additional gastrectomy required for elderly patients after endoscopic submucosal dissection with endoscopic curability C-2 for early gastric cancer?. Digestion.

[103] Scotte F., Bossi P., Carola E., Cudennec T., Dielenseger P., Gomes F., Knox S., Strasser F. (2018). Addressing the quality of life needs of older patients with cancer: A SIOG consensus paper and practical guide. Ann. Oncol..

[104] Wildiers H., Heeren P., Puts M., Topinkova E., Janssen-Heijnen M.L., Extermann M., Falandry C., Artz A., Brain E., Colloca G. (2014). International Society of Geriatric Oncology consensus on geriatric assessment in older patients with cancer. J. Clin. Oncol..

[105] Ishihara R., Matsuura N., Hanaoka N., Yamamoto S., Akasaka T., Takeuchi Y., Higashino K., Uedo N., Iishi H. (2017). Endoscopic imaging modalities for diagnosing invasion depth of superficial esophageal squamous cell carcinoma: A systematic review and meta-analysis. BMC Gastroenterol..

[106] Ishihara R., Mizusawa J., Kushima R., Matsuura N., Yano T., Kataoka T., Fukuda H., Hanaoka N., Yoshio T., Abe S. (2021). Assessment of the diagnostic performance of endoscopic ultrasonography after conventional endoscopy for the evaluation of esophageal squamous cell carcinoma invasion depth. JAMA Netw. Open.

[107] Kato M., Uedo N., Nagahama T., Yao K., Doyama H., Tsuji S., Gotoda T., Kawamura T., Ebi M., Yamamoto K. (2019). Self-study of the non-extension sign in an e-learning program improves diagnostic accuracy of invasion depth of early gastric cancer. Endosc. Int. Open.

[108] Li X., Zhu M., Wang Y., Niu Y., Ji M., Li P., Zhang S. (2021). Diagnostic efficacy and decision-making role of preoperative endoscopic ultrasonography in early gastric cancer. Front. Med..

[109] Kuroki K., Oka S., Tanaka S., Yorita N., Hata K., Kotachi T., Boda T., Arihiro K., Chayama K. (2021). Clinical significance of endoscopic ultrasonography in diagnosing invasion depth of early gastric cancer prior to endoscopic submucosal dissection. Gastric Cancer.

[110] Zhu Y., Wang Q.C., Xu M.D., Zhang Z., Cheng J., Zhong Y.S., Zhang Y.Q., Chen W.F., Yao L.Q., Zhou P.H. (2019). Application of convolutional neural network in the diagnosis of the invasion depth of gastric cancer based on conventional endoscopy. Gastrointest. Endosc..

